# Prognostic value of granulocyte colony-stimulating factor in patients with non-metastatic clear cell renal cell carcinoma

**DOI:** 10.18632/oncotarget.19540

**Published:** 2017-07-25

**Authors:** Zheng Liu, Yu Zhu, Yiwei Wang, Qiang Fu, Hangcheng Fu, Zewei Wang, Junyu Zhang, Gaoxiang Li, Jiejie Xu, Bo Dai

**Affiliations:** ^1^ Department of Urology, Fudan University Shanghai Cancer Center, Shanghai, China; ^2^ Department of Oncology, Shanghai Medical College, Fudan University, Shanghai, China; ^3^ Department of Urology, Ninth People’s Hospital, Shanghai Jiao Tong University School of Medicine, Shanghai, China; ^4^ Department of Biochemistry and Molecular Biology, School of Basic Medical Sciences, Fudan University, Shanghai, China

**Keywords:** carcinoma, renal cell, granulocyte colony-stimulating factor, prognosis, neoplasm recurrence

## Abstract

Granulocyte colony-stimulating factor is a well-known cytokine to stimulate inflammatory cells. We sought to investigate the prognostic value of its expression in patients with non-metastatic clear cell renal cell carcinoma. Enrolled in this study were 228 eligible patients treated with curative nephrectomy for clear cell renal cell carcinoma during 2008. Granulocyte colony-stimulating factor expression was detected by immunohistochemistry in patient specimens, and was divided into three groups according to the distribution of its immunohistochemistry score. Subgroup analyses were performed to evaluate its risk stratification ability. Cox regression models were applied to analyze the impact of prognostic factors. We found that high granulocyte colony-stimulating factor expression was associated with diminished recurrence-free survival (*P*<0.001). Its expression had stronger stratification ability in late disease patients, and was further identified as an independent prognosticator for recurrence-free survival. Moreover, nomogram based on granulocyte colony-stimulating factor expression presented a better prognostic ability compared with current prognostic systems (the concordance index = 0.874). To conclude, intratumoal granulocyte colony-stimulating factor expression could be a potential prognosticator for recurrence-free survival in non-metastatic clear cell renal cell carcinoma patients. Incorporating its expression into other pathologic factors provided a finer individual model for non-metastatic clear cell renal cell patients.

## INTRODUCTION

According to the latest statistics [1], renal cell carcinoma (RCC) accounts for 62,700 new cancer cases in the US and 66800 in China [2]. A study from Europe [3] unveiled the majority of increased RCC cases from 1984 to 2010 were non-metastatic tumors, and most of them were clear cell RCC (ccRCC). Despite the advances in diagnosis and technics of nephrectomy, nearly one-third patients undergoing nephrectomy still inevitably experienced recurrence or progressed to metastasis, and finally to incurable disease [4]. In ccRCC, even though patients have similar clinicopathologic features, their clinical outcomes may be entirely different [5]. The unpredictable natural history of RCC obstructed estimation accuracy on prognosis of patients. Therefore, improved prognosticators are needed urgently.

TNM stage and Fuhrman grade remain the mainstream prognosticators for RCC patients. Three integrated prognostic prediction models: University of California Integrated Staging System (UISS), Mayo Clinic stage, size, grade, and necrosis (SSIGN) score and Leibovich score [6, 7] are also widely used in prognostic prediction. These models may have a potential for further improvement of accuracy via the corporation of different biomarkers [7].

Currently, many biomarkers focusing on tumor microenvironment have been investigated in combination with those models, including intratumoral neutrophils [8, 9]. Granulocyte colony-stimulating factor (G-CSF) is the primary cytokine that activates the proliferation and differentiation of myeloid progenitors, and promotes neutrophil release from bone marrow [10]. Though G-CSF has been long considered to be secreted by hemocytes, recent studies showed that G-CSF also could be produced by non-hematopoietic malignancies, such as lung cancer cells, bladder cancer cells and even RCC cells [11–13]. Additionally, Waight et al found that G-CSF acted as a key role in granulocytic myeloid-derived suppressor cells (MDSC) accumulation, and then promoted the tumor growth [14]. Considering these studies, G-CSF may be a latent regulator of tumor microenvironment. Thus, the possible prognostic prediction ability of G-CSF needs to be explored.

In this study, we tried to investigate the potential role of G-CSF in the prognosis of ccRCC patients. G-CSF expression was evaluated by immunohistochemistry staining in ccRCC tissues; its correlation with clincopathologic features and clinical outcome of patients was assessed. We further evaluated whether G-CSF expression could refine current prognostic models. Moreover, nomogram based on the G-CSF expression and several other well-known pathologic features was established, and its prognostic value was analyzed.

## RESULTS

### Patient characteristics and associations with G-CSF expression

**Figure 1:**
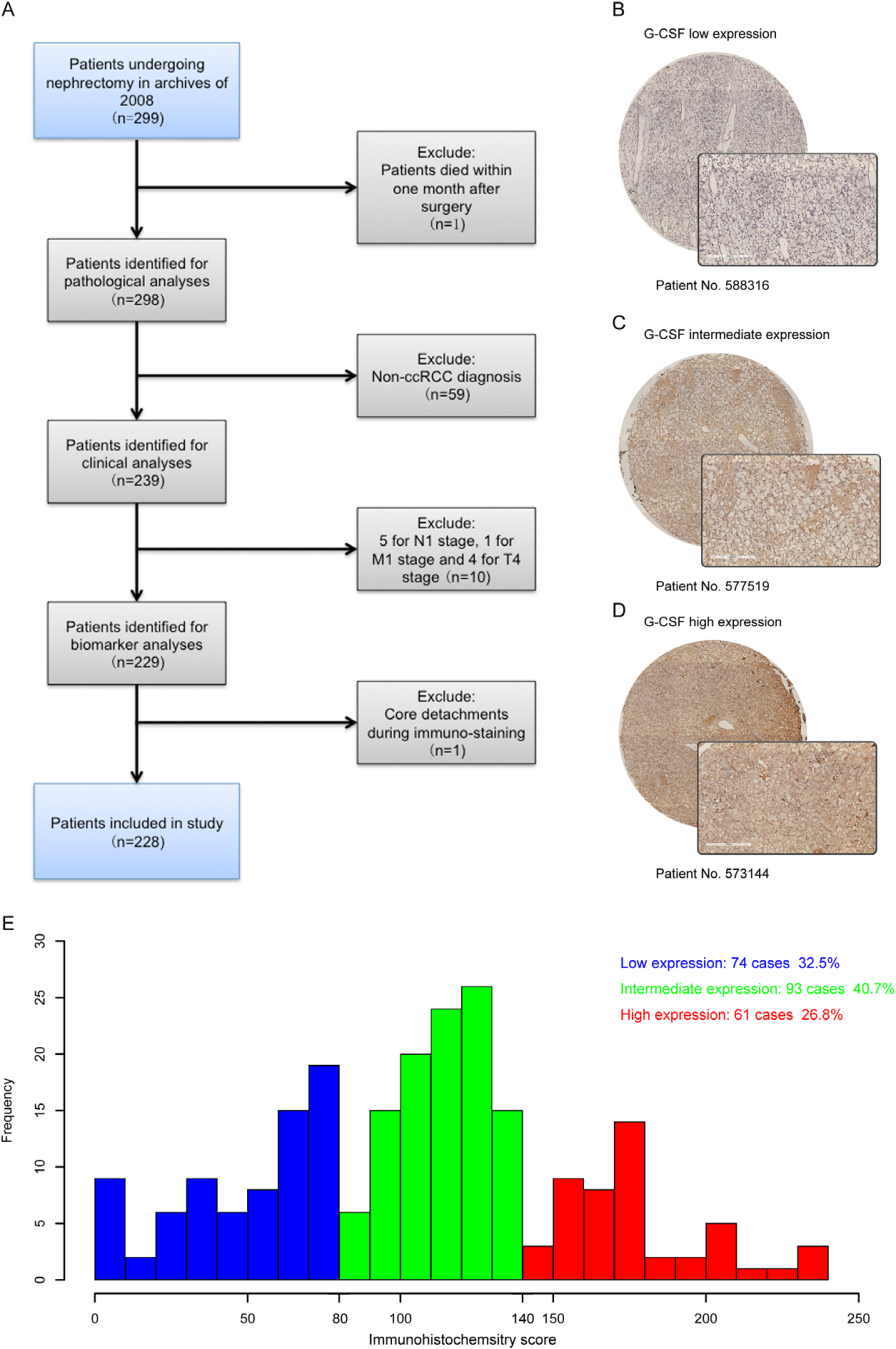
G-CSF expression in ccRCC tissues and the result of immunohistochemistry score **(A)** Flowchart of study patients’ inclusion. **(B-D)** Representative photographs of (B) low, (C) intermediate and (D) high G-CSF immunostaining in ccRCC tissues (original magnification ×200). **(E)** Frequency distribution of G-CSF immunohistochemistry score in 228 ccRCC tumor samples, showing tertiles of 80 and 140 used to divide low, intermediate and high expression subgroups.

The clinicopathologic characteristics of 228 eligible patients were summarized in Table 1. G-CSF positive staining was mainly located in the cytoplasm of ccRCC cells. Representative pictures of low, intermediate and high expression of G-CSF were illustrated in Figure 1B, 1C and 1D, respectively. According to the distribution of immunohistochemistry score, tertile scores 80 and 140 were determined as the cut-off values, which separated the population into 74 patients with low G-CSF expression, 93 patients with intermediate expression, and 61 patients with high expression (Figure 1E).

**Table 1:** Patient characteristics and associations with G-CSF expression

Factor	Patients		G-CSF expression			
	No.	%	Low (n=74)	Intermediate (n=93)	High (n=61)	*P*
Age at surgery (year)						0.477*
Median (IQR)	56 (48-62)		54 (47-61)	56 (49-63)	57 (46-66)	
Gender						0.524†
Male	170	74.6	52	70	48	
Female	58	25.4	22	23	13	
Tumor size (cm)						0.190*
Median (IQR)	4.0 (3.0-5.5)		3.4 (2.5-5.0)	3.0 (4.0-6.0)	4.0 (3.0-6.0)	
T stage						0.023‡
T1	148	64.9	56	57	35	
T2	25	11.0	6	11	8	
T3	55	24.1	12	25	18	
Fuhrman grade						<0.001‡
1	47	20.6	28	17	2	
2	95	41.7	31	47	17	
3	57	25.0	13	16	28	
4	29	12.7	2	13	14	
Tumor necrosis						0.113†
Absent	183	80.3	64	75	44	
Present	45	19.7	10	18	17	
UISS						<0.001‡
Low risk	93	40.8	42	39	12	
Intermediate risk	121	53.1	31	47	43	
High risk	14	6.1	1	7	6	
SSIGN score						0.005‡
0-3	152	66.7	57	61	34	
4-7	70	30.7	17	30	23	
≥8	6	2.6	0	2	4	
Leibovich score						<0.001‡
0-2	115	50.4	45	46	24	
3-5	90	39.5	29	39	22	
≥6	23	10.1	0	8	15	
Follow-up (month)						<0.001*
Median (IQR)	73 (66-74)		73 (72-74)	73 (67-74)	69 (57-73)	
Events						
Recurrence	43	18.9	5	16	22	<0.001†

G-CSF: granulocyte colony-stimulating factor; IQR: interquartile range; UISS: UCLA Integrated Staging System; SSIGN: stage, size, grade and necrosis.

*Kruskal-Wallis test

†Wilcoxon rank-sum test

‡Spearman’s rank correlation

Table 1 presented associations between the G-CSF expression and patient characteristics. Higher G-CSF expression was associated with more advanced T stage and Fuhrman grade (*P*=0.023 and *P*<0.001, respectively). Patients with higher expression of G-CSF were more likely to experience recurrence (*P*<0.001), and appear in a worse classification group in UISS (*P*<0.001), SSIGN (*P*=0.005), and Leibovich score (*P*<0.001). Although in this study population, the number of male patients (74.6%) was nearly triple than female patients (25.4%), G-CSF expression was not associated with gender (*P*=0.524). In addition, G-CSF expression was not associated with age of patients (*P*=0.477); and found to have no significant correlation with tumor size (*P*=0.190), albeit increased G-CSF expression tended to present a larger tumor size.

### Expression of G-CSF further stratified late disease patients

**Figure 2:**
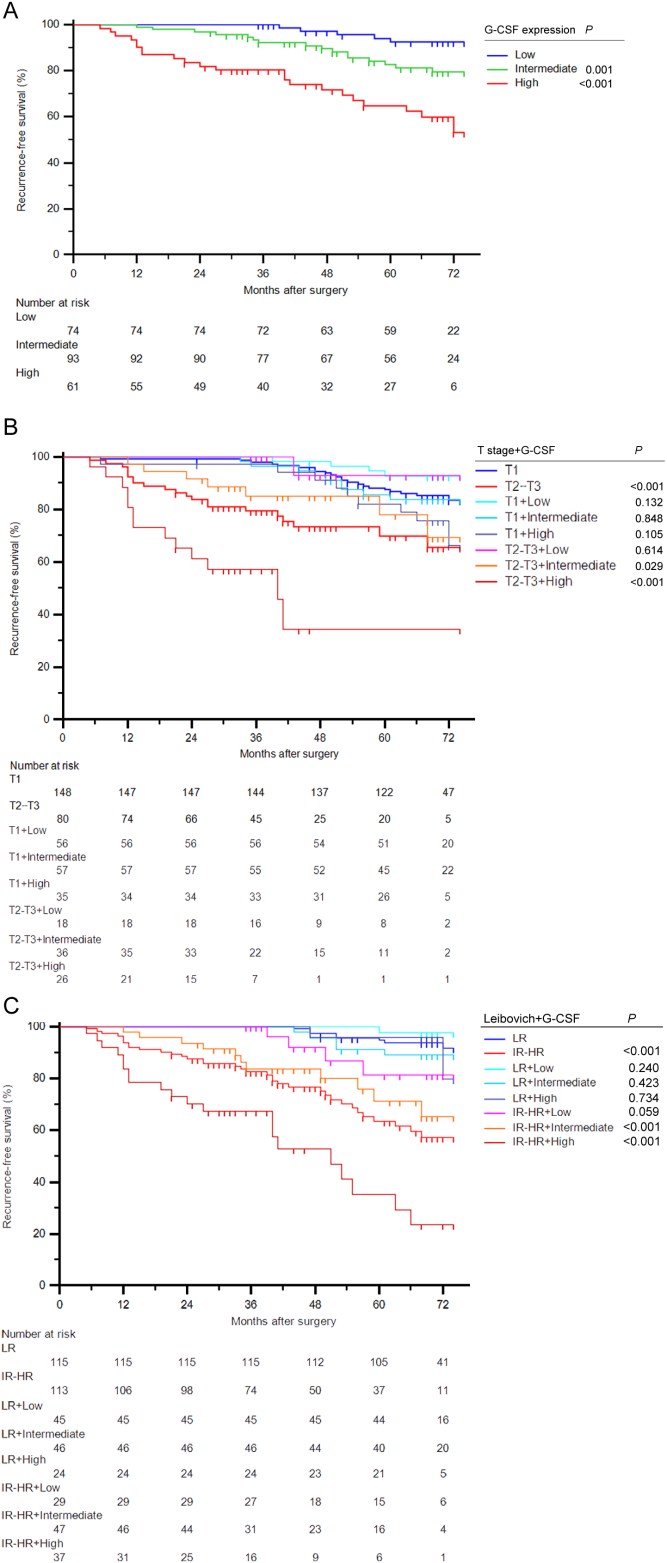
Stratification ability for RFS of G-CSF expression in ccRCC patients **(A)** Kaplan-Meier curve for RFS of ccRCC patients according to G-CSF expression. **(B-C)** Kaplan-Meier curves for RFS of ccRCC patients combined G-CSF expression with (B) T stage and (C) Leibovich score. Log-rank test *P* values.

**Figure 3:**
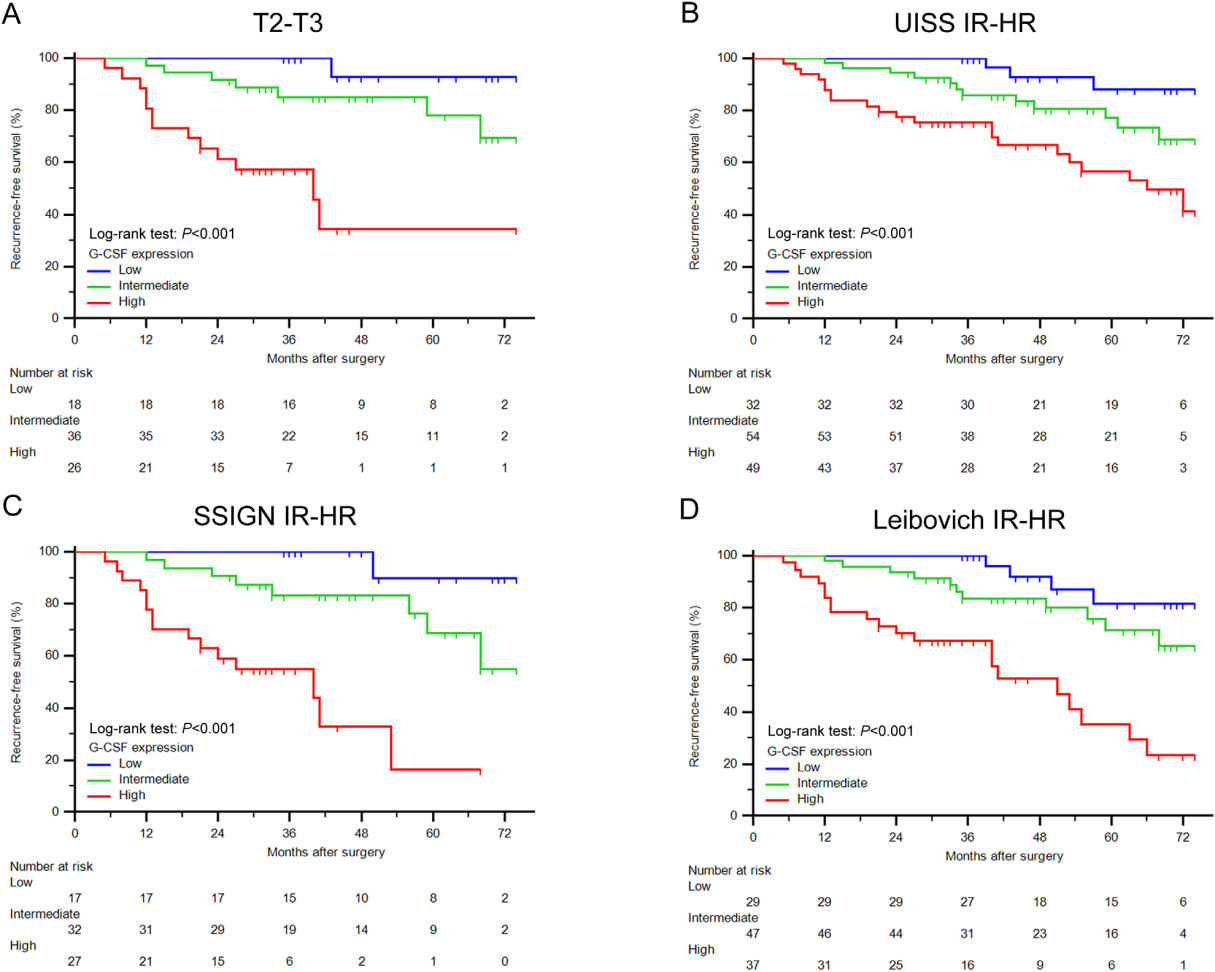
Survival analyses for RFS of ccRCC patients within different risk groups **(A-D)** Kaplan-Meier curves for RFS of ccRCC patients categorized in (A) T2-T3, (B) UISS intermediate and high risk, (C) SSIGN intermediate and high risk, and (D) Leibovich intermediate and high risk group according to G-CSF expression. Log-rank test *P* values.

Kaplan-Meier survival analyses were applied to evaluate RFS according to G-CSF expression groups. Patients with high and intermediate G-CSF expression had a significant shorter RFS (*P*<0.001 and *P*=0.001, respectively) than those with low expression (Figure 2A). Further survival analyses confirmed that G-CSF expression could further stratified recurrence risks of T2-T3 patients and intermediate and high-risk group patients according to UISS, SSIGN and Leibovich score (Figure 3A, 3B, 3C and 3D, respectively). However, this stratification ability was not observed in early disease patients (Supplementary Figure 1).

To further explore the prognostic value of G-CSF expression, we conducted subgroup analyses combined various risk groups with G-CSF. As illustrated in Figure 2B, G-CSF expression could further stratified patients grouped by T stage. The RFS rate at the last follow-up of T2-T3 patients with low G-CSF expression (92.9%) had no significant difference with T1 patients (83.7%) (*P*=0.614). Notably, T2-T3 patients had a 65.5% RFS rate at the last follow-up, once these patients presented high G-CSF expression, their RFS rate would remarkably dropped to 34.3% (*P*=0.003). Similarly, in Figure 2C, patients were separated into different risk groups according to Leibovich score. Among these patients, intermediate and high-risk patients with low G-CSF expression had a relatively close RFS rate (81.5%) compared with low-risk patients (91.6%) (*P*=0.059). Meanwhile, intermediate and high-risk patients with high G-CSF expression had a nearly half RFS rate (23.5%) than original risk group patients (57.4%) at the last follow-up (*P*=0.001). These similar findings were also observed in patients grouped by UISS and SSIGN score (Supplementary Figure 2A and 2B, respectively).

Thus, these results demonstrated that even if patients were already stratified in T2-T3 or intermediate and high-risk group, these patients with low expression of G-CSF would experience a relative close RFS compared with T1 or low-risk patients. High expression of G-CSF would also indicate a more severe unfavorable RFS within late stage patients.

### G-CSF expression was an independent prognosticator for RFS

To evaluate the independence of G-CSF expression prognostic ability, univariate and multivariate analyses were conducted. As listed in Table 2, high G-CSF expression was significantly associated with worse RFS in univariate analyses (HR: 7.745, 95%CI: 2.927-20.492, *P*<0.001). After a 1000-resampled bootstrap multivariate analyze, its significance remained (HR: 6.123, 95%CI: 2.596-21.780, *P*=0.001). In company with tumor size, T stage, Fuhrman grade and tumor necrosis, G-CSF expression was also verified as an independent prognosticator of RFS for non-metastatic ccRCC patients.

**Table 2:** Univariate and multivariate Cox regression analyses of potential prognostic factors for recurrence-free survival

Factor	Univariate analyses		Multivariate analyses†	
	HR (95% CI)	*P*	HR (95% CI)	*P*
Age at surgery (year)	1.023 (0.996-1.051)	0.096	Adjusted	
Gender (male vs. female*)	1.084 (0.534-2.199)	0.824	Adjusted	
Tumor size (cm)	1.433 (1.287-1.595)	<0.001	1.355 (1.206-1.537)	0.001
T stage (II+III vs. I*)	3.407 (1.838-6.315)	<0.001	2.293 (1.021-4.870)	0.020
Fuhrman grade		<0.001		0.007
3 vs. 1+2*	2.906 (1.426-5.922)	0.003	1.752 (0.812-4.039)	0.121
4 vs. 1+2*	9.650 (4.454-20.909)	<0.001	4.735 (2.083-11.246)	0.002
Tumor necrosis (present vs. absent*)	4.052 (2.187-7.507)	<0.001	3.951 (2.073-7.668)	0.001
G-CSF expression		<0.001		0.002
intermediate vs. low*	2.937 (1.075-8.020)	0.036	2.328 (0.995-8.516)	0.071
high vs. low*	7.745 (2.927-20.492)	<0.001	6.123 (2.596-21.780)	0.001

HR: hazard ratio; CI: confidence interval; G-CSF: granulocyte colony-stimulating factor.

*Reference group

†Calculated on the basis of adjusted survival function for age and gender by the time of surgery. Bootstrapping with 1000 resamples were used.

### A nomogram based on G-CSF expression and comparison with current models

Combined with other known pathologic variables from the validated regression models (Table 2), a nomogram based on G-CSF expression was constructed (Supplementary Figure 3A). Calibration plot of the nomogram indicated that the performance was close to the ideal prediction (Supplementary Figure 3B). In Table 3, we compared predictive accuracy of G-CSF nomogram with TNM, UISS and SSIGN systems within T2-T3 patients via C-indices. G-CSF based nomogram had a better c-index (0.874) and a lower AIC (136.5), indicating a better prognostic ability than conventional clinicopathologic variable based models, especially among late disease patients.

**Table 3:** Comparison of prognostic accuracies of the Nomograms based on the G-CSF expression, UISS, SSIGN and Leibovich scoring system in pT2-3 population

Recurrence-free survival	C-index	*P* value	AIC
Nomogram*	0.874		136.5
UISS	0.614	<0.01	166.5
SSIGN	0.711	<0.01	155.3
Leibovich	0.708	<0.01	156.4

G-CSF: granulocyte colony-stimulating factor; UISS: UCLA Integrated Staging System; SSIGN: stage, size, grade and necrosis; AIC: Akaike's information criterion.

C-indices are calculated from 1000 bootstrap samples to protect from overfitting.

*Reference group

## DISCUSSION

G-CSF is a well-known cytokine involved in differentiation, proliferation and activation of granulocytes [10]. Conventionally, in oncotherapy filed, G-CSF was applied as adjuvant chemotherapy in various types of leukemia, and as a remedy to side effects, such as febrile neutropenia, caused by chemotherapy in some solid tumors [15, 16]. Notwithstanding, opposite to current clinical use, newly studies presented that G-CSF may act as a tumor promoter in various cancers. In head and neck squamous cell carcinoma, G-CSF was reported to enhance tumor invasion and metastasis via the recruitment of inflammatory cells [17]. Similar findings were also demonstrated in lung metastasis [12]. In addition, recent basic studies proved that except for the mobilization of granulocytes, G-CSF might function in more complicated ways. For instance, G-CSF was reported to promote survival and growth of bladder cancer cells by stimulating STAT3-depedent survivn expression [13], and to potentiate tumor progression through its neurotrophic ability on nerve in prostate cancer [18]. Consistent with these results, we also demonstrated that high intratumoral G-CSF expression was an independent prognosticator of diminished RFS for non-metastatic ccRCC. Furthermore, high expression of G-CSF was also associated with advanced pathologic features and high-risk group in our study. Thus, G-CSF may play a more complicated and significant role in tumor progression than traditional thoughts.

In ccRCC, we have previously reported that high macrophage colony-stimulating factor (M-CSF) and high granulocyte macrophage colony-stimulating factor (GM-CSF) could predict worse clinical outcomes of ccRCC patients [19, 20]. Colony stimulating factor may stimulate various immune cells and thus to educate the tumor microenvironment. As one of the colony-stimulating factors, G-CSF could activate granulocytes, especially neutrophils [10]. However, tumor infiltrated neutrophils, or tumor-associated neutrophils (TAN), were reported to have two polarization phenotypes: the anti-tumor N1 type and the pro-tumor N2 type [21]. A recent study indicated that intratumoral neutrophil in ccRCC was a negative prognostic predictor in patient outcome [8], which was in accordance with the pro-tumor feature of G-CSF in our study. In peripheral blood, increased neutrophils and neutrophil-lymphocyte ratio (NLR) was also reported as an indicator of poor prognosis in RCC [22–24]. As well as activating neutrophils, G-CSF was revealed to be responsible for accumulation of granulocytic MDSC, and lead to the immunosuppression and tumor growth [14]. These intratumoral inflammatory cells with other inflammatory factors besides G-CSF could then form tumor microenvironment and promote tumor growth per se [25, 26]. Taken these studies, as a primary cytokine to stimulate both tissue and serum based neutrophils, and a critical inflammatory element in accumulation of granulocytic MDSC, G-CSF was likely to be a general and accurate prognosticator in ccRCC.

Early stage (T1) RCC patients who underwent nephrectomy have a favorable 83% 5-year cancer specific survival rate. Surprisingly, these figures dropped dramatically to 42% for T3 and 28% for T4, giving advanced stage patients a bleak outcome [27]. Refined prognostic models for ccRCC patients are urgently wanted for either identifying high-risk postoperative patients for more active surveillance or avoiding unnecessary frequent follow-up with imaging for low-risk patients [28]. However, current primary prognostic models, such as TNM stage, UISS and SSIGN are generally based on clinicopathologic features. Components of tumor microenvironment, which also have an important role in tumor development and progression, are not reflected in these systems. For this reason, it is quite possible that incorporation of G-CSF expression into these established models would sharpen their prognostic ability. Our results indicated that ccRCC patients with intermediate and late disease might face different prognosis according to G-CSF expression in tumor specimens. Although further external validation were required, this study might benefit low G-CSF expression patients, for they would have a similar RFS compared to low-risk patients, even they were categorized as intermediate and late disease according to various conventional prognosis models. While low G-CSF would rescue them from intensive surveillance, high G-CSF expression indicated an even worse clinical outcome though they were already in advanced stage. Thus, G-CSF expression could provide additional tumor molecular information to current pathological based prognostic models. Furthermore, a nomogram incorporated with G-CSF expression and other pathologic factors was also constrcued. Of note, compared with those original prognostic models, our study had multiple differences in follow-up time, sample size and characteristics of study population. These variances caused the difference in absolute c-index value between our study and others [29]. To diminish this confounding factor, we treated c-index as a comparative variable instead of an absolute value. As listed in Table 3, the constructed nomogram displayed a significant better RFS predictive ability than current mainstream models.

The main limitations of our study are its retrospective study design and lack of external validation. Thus, this study still needs to be replicated and external validated independently. Moreover, the median follow-up time in this study was 73 months, which was insufficient to calculate 10-year RFS. Longer observation was required to compare long-term prognostic value of this model with other pathological based models. Relatively small cores from microarrays may not be fully representative of the whole tumor tissue, and this intratumoral heterogeneity may weaken the robustness of the predictive ability of this prognosticator. Finally, the specific molecular mechanism of G-CSF in ccRCC needs further explored.

To conclude, our study demonstrated that high intratumoral G-CSF expression could be a novel independent adverse prognosticator in non-metastatic ccRCC patients. Incorporation of G-CSF expression into current prognostic models, such as T stage, UISS, SSIGN and Leiboivch score could refine their prognostic ability. Low G-CSF expression in late disease patients suggests a relatively similar RFS compared with low-risk patients. Nomogram combined G-CSF expression and other conventional pathological factors has a better prognosis predictive performance in T2-T3 patients than other models.

## MATERIALS AND METHODS

### Patients

Approved by institution review board, we retrospectively identified 299 consecutive patients who underwent a curative-intended nephrectomy for non-metastatic RCC at Fudan University Shanghai Cancer Center during the year 2008. Written informed consent was obtained from each patient. Selection criteria were as follows: (1) no history of any anti-tumor therapy; (2) no history of other malignant tumors; (3) histopathologically proven ccRCC. Detailed procedures of patient recruitment were illustrated in Figure 1A. Patients were followed up postoperatively with physical examinations, laboratory studies, chest imaging and abdominal ultrasounds or CT scans every 6 months for the first 2 years and annually thereafter for 5 years. The endpoint of interest was recurrence-free survival (RFS), and was calculated from the date of nephrectomy to the date of recurrence, or to the date of the last follow-up.

The median follow-up period was 73 months (range: 39-74 months). At the time of last follow-up, 43 patients (18.9%) had experienced recurrence. For each patient, the following clinicopathologic information was collected: age, gender, tumor size, T stage, Fuhrman grade and presence of histologic tumor necrosis. All original hematoxylin and eosin slides were centrally reviewed by one experienced genitourinary pathologist (C. Zhai) to obtain pathologic features. Patients were staged using radiographic reports and postoperative pathological and were reassigned according to the 2010 AJCC TNM classification [30]. Since Fuhrman grades 1 and 2 have a similar contribution to clinical outcome in ccRCC according to most prognostic systems, such as SSIGN and Leibovich score [6, 7], we combined cases of grade 1 with grade 2 in the following analyses. The UISS system, SSIGN score and Leibovich score classified all patients into three different risk categories, respectively.

### Immunohistochemistry and scoring

Tissue microarray construction and immunohistochemistry protocol were previously described [31]. The primary antibody against human G-CSF (ab112112, Abcam; dilution 1:50) was applied in the procedure. A semiquantitative score on a scale of 0 to 300 was calculated for each sample by multiplying the staining intensity (0, negative staining; 1, weak; 2, moderate; and 3, strong) and the percentage of cells (0%-100%) at each intensity level. An experienced urology pathologist (C. Zhai) evaluated the staining without knowledge of patient outcome. The mean score of the duplicate tissue spots from each patient was used for statistical analyses. The kappa-value between the 2 sets of scores was 0.83, indicating a good concordance.

### Statistical analyses

Although G-CSF expression was recorded as a continuous variable, in an attempt to simplify the interpretation of the association with pathologic factors and patient outcome, we divided G-CSF expression into low, intermediate and high group at the tertile scores according to the distribution of immunohistochemistry score (Figure 1E). Association between G-CSF expression levels and clincopathlogic features was analyzed with Wilcoxon rank-sum test for unordered categorical variables, Spearman’s rank correlation for ordered categorical variables and Kruskal-Wallis *H* test for continuous variables. Kaplan-Meier method with log-rank test was applied to establish and compare survival curves. The Cox proportional hazards regression model was applied to perform univariate and multivariate analyses, and bootstrapping with 1000 resamples were used. The predictive accuracy of different prognostic models was quantified by the Harrell concordance index (*c*-index), which ranges from 0.5 (no predictive power) to 1 (perfect prediction). Akaike’s information criterion (AIC) was also calculated. Established nomogram was validated by 200 bootstrap resamples to decrease overfit bias, and its performance was explored graphically within a calibration plot.

Data were analyzed using SPSS 20.0 and Stata 13.0. Medcalc software was used to plot the survival curves and R software (version 3.2.1, the ‘rms’ package) was used to build the nomograms. Reported recommendations for tumor marker prognostic studies (REMARK) [32] criteria were obeyed throughout the study (Supplementary Table 1).

## SUPPLEMENTARY MATERIALS FIGURES AND TABLE





## Abbreviations

RCC: renal cell carcinoma
ccRCC: clear cell renal cell carcinoma
UISS: University of California Integrated Staging System
SSIGN: Mayo Clinic stage, size, grade, and necrosis
G-CSF: granulocyte colony-stimulating factor
MDSC: myeloid-derived suppressor cells
RFS: recurrence-free survival
AIC: Akaike’s information criterion
HR: hazard ratio
CI: confidence interval
