# Supplementary material for: Prognostic value of granulocyte colony-stimulating factor in patients with non-metastatic clear cell renal cell carcinoma
Source: Oncotarget. 2017 Jul 25;8(41):69961–71. doi: 10.18632/oncotarget.19540 (PMC5642530; doi:10.18632/oncotarget.19540)
Supplement: Supplementary file 1 [file oncotarget-08-69961-s001.pdf]

# Prognostic value of granulocyte colony-stimulating factor in patients with non-metastatic clear cell renal cell carcinoma

## SUPPLEMENTARY MATERIALS

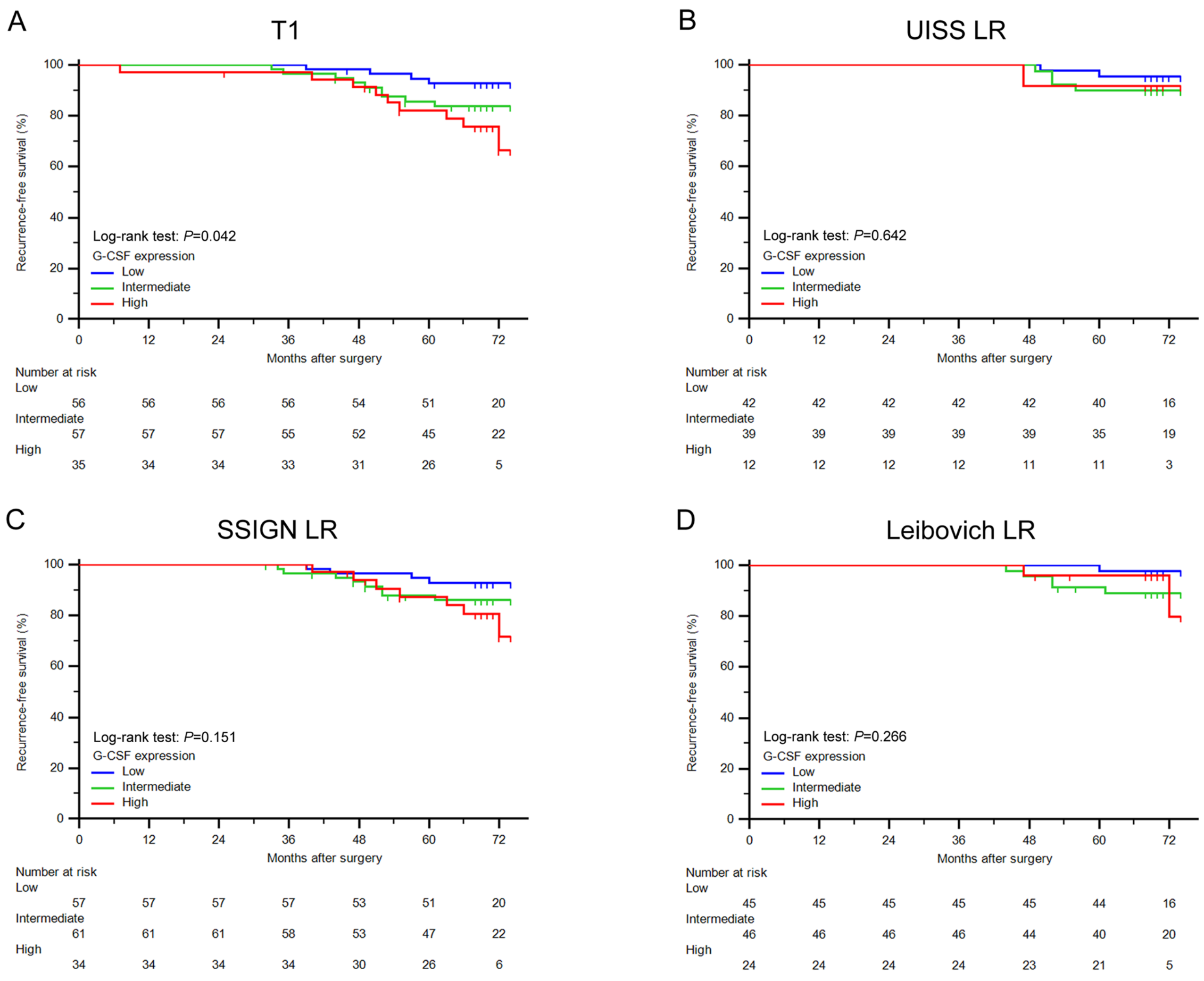


**Supplementary Figure 1: Survival analyses for RFS of ccRCC patients within different risk groups. (A-D)** Kaplan-Meier curves for RFS of ccRCC patients categorized in (A) T1, (B) UISS low risk, (C) SSIGN low risk, and (D) Leibovich low risk group according to G-CSF expression. Log-rank test *P* values.

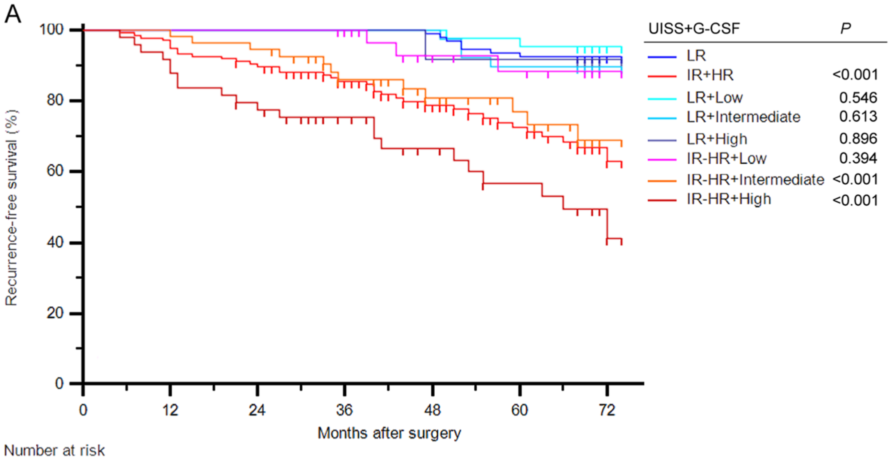


Number at risk

| | 0 | 12 | 24 | 36 | 48 | 60 | 72 |
|---|---|---|---|---|---|---|---|
| LR | 93 | 93 | 93 | 93 | 92 | 86 | 38 |
| IR+HR | 135 | 128 | 120 | 96 | 70 | 56 | 14 |
| LR+Low | 42 | 42 | 42 | 42 | 42 | 40 | 16 |
| LR+Intermediate | 39 | 39 | 39 | 39 | 39 | 35 | 19 |
| LR+High | 12 | 12 | 12 | 12 | 11 | 11 | 3 |
| IR-HR+Low | 32 | 32 | 32 | 30 | 21 | 19 | 6 |
| IR-HR+Intermediate | 54 | 53 | 51 | 38 | 28 | 21 | 5 |
| IR-HR+High | 49 | 43 | 37 | 28 | 21 | 16 | 3 |

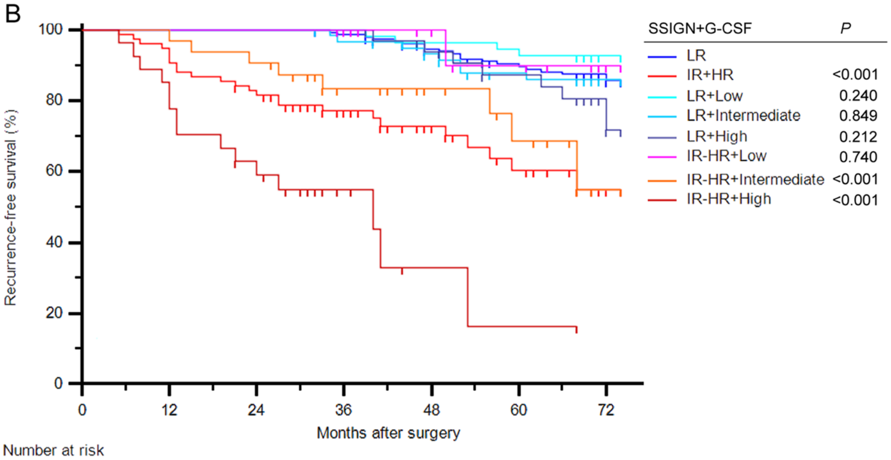


Number at risk

| | 0 | 12 | 24 | 36 | 48 | 60 | 72 |
|---|---|---|---|---|---|---|---|
| LR | 152 | 152 | 152 | 149 | 136 | 124 | 48 |
| IR+HR | 76 | 69 | 61 | 40 | 26 | 18 | 4 |
| LR+Low | 57 | 57 | 57 | 57 | 53 | 51 | 20 |
| LR+Intermediate | 61 | 61 | 61 | 58 | 53 | 47 | 22 |
| LR+High | 34 | 34 | 34 | 34 | 30 | 26 | 6 |
| IR-HR+Low | 17 | 17 | 17 | 15 | 10 | 8 | 2 |
| IR-HR+Intermediate | 32 | 31 | 29 | 19 | 14 | 9 | 2 |
| IR-HR+High | 27 | 21 | 15 | 6 | 2 | 1 | 0 |

**Supplementary Figure 2: Stratification ability for RFS of G-CSF expression in ccRCC patients. (A-B)** Kaplan-Meier curves for RFS of ccRCC patients combined G-CSF expression with (A) UISS and (B) SSIGN score. Log-rank test *P* values.

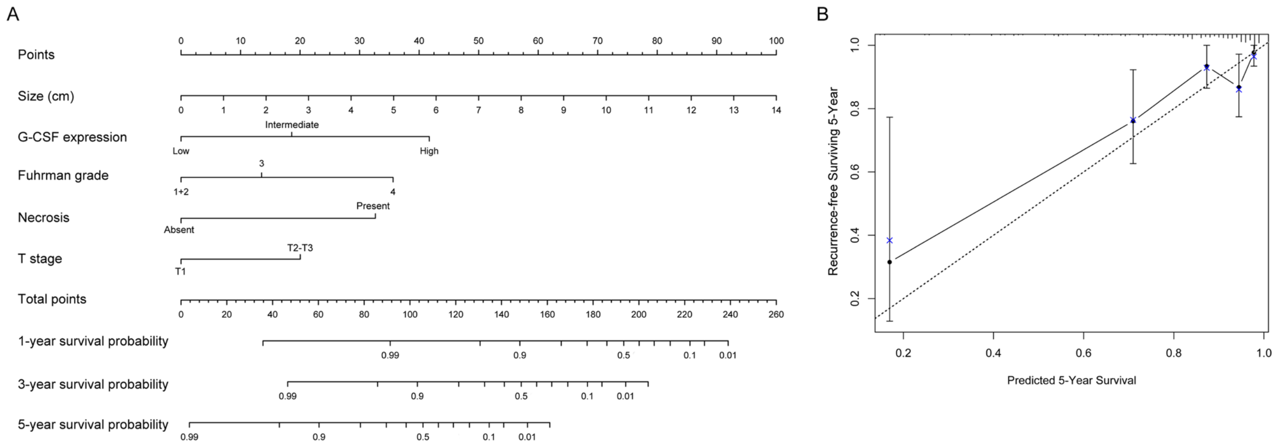


**Supplementary Figure 3: Nomogram established for the prediction of RFS in patients with non-metastasis ccRCC.** **(A)** Nomogram predicting RFS in non-metastasis ccRCC patients after surgery. Draw an upward vertical line from the covariate to the point bar to calculate points. Based on the sums of the covariate points, draw a downward vertical line from the total points line to calculate RFS. **(B)** Calibration curve of the nomogram for 5-year RFS. Dotted lines represent the performance of an ideal nomogram. Solid lines represent the performance of the proposed nomogram. Error bars represent 95% CI.

**Supplementary Table 1: Adherence to REMARK guidelines**

See Supplementary File 1
