# Supplementary material for: Prognostic value of granulocyte colony-stimulating factor in patients with non-metastatic clear cell renal cell carcinoma
Source: Oncotarget. 2017 Jul 25;8(41):69961–71. doi: 10.18632/oncotarget.19540 (PMC5642530; doi:10.18632/oncotarget.19540)
Supplement: Supplementary file 2 [file oncotarget-08-69961-s002.doc]

**Supplementary Table 1: Adherence to REMARK guidelines**

| **REMARK checklist** | **This manuscript** |
| --- | --- |
| **Introduction** |  |
| 1. State the marker examined, the study objectives, and any pre-specified hypotheses. | Intratumoral G-CSF expression in ccRCC; its prognostic potential for RFS; and G-CSF could refine current prognostic models. (Details in **Introduction** section) |
| **Materials and Methods** |  |
| ***Patients*** |  |
| 2. Describe the characteristics (for example, disease stage or co-morbidities) of the study patients, including their source and inclusion and exclusion criteria. | Non-metastatic ccRCC, details in **Patients** part. |
| 3. Describe treatments received and how chosen (e.g., randomized or rule-based). | Details in **Patients** part. |
| ***Specimen characteristics*** |  |
| 4. Describe type of biological material used (including control samples) and methods of preservation and storage. | Details in **Immunohistochemistry and scoring** part. |
| ***Assay methods*** |  |
| 5. Specify the assay method used and provide (or reference) a detailed protocol, including specific reagents or kits used, quality control procedures, reproducibility assessments, quantitation methods, and scoring and reporting protocols. Specify whether and how assays were performed blinded to the study endpoint. | Details in **Immunohistochemistry and scoring** part. |
| ***Study design*** |  |
| 6. State the method of case selection, including whether prospective or retrospective and whether stratification or matching (e.g. by stage of disease or age) was employed. Specify the time period from which cases were taken, the end of the follow-up period, and the median follow-up time. | Retrospective study, non-metastatic ccRCC cases were selected. Details in **Patients** part and **Patient characteristics and associations with G-CSF expression** part. |
| 7. Precisely define all clinical end points examined. | Details in **Patients** part. |
| 8. List all candidate variables initially examined or considered for inclusion in models. | Listed in **Table 1**. |
| 9. Give rationale for sample size; if the study was designed to detect a specified effect size, give the target power and effect size. | As listed in **Table 1**, each variable studied had at least 25 events. |
| ***Statistical analysis methods*** |  |
| 10. Specify all statistical methods, including details of any variable selection procedures and other model-building issues, how model assumptions were verified, and how missing data were handled. | Details in **Statistical analyses** part. |
| 11. Clarify how marker values were handled in the analyses; if relevant, describe methods used for cut point determination. | Details in **Statistical analyses** part. |
| **Results** |  |
| ***Data*** |  |
| 12. Describe the flow of patients through the study, including the number of patients included in each stage of the analysis (a diagram may be helpful) and reasons for dropout. Specifically, both overall and for each subgroup extensively examined report the numbers of patients and the number of events. | Listed in **Patients** part, **Table 1**, **Figure 1**. |
| 13. Report distributions of basic demographic characteristics (at least age and sex), standard (disease-specific) prognostic variables, and tumor marker, including numbers of missing values. | Listed in **Table 1**. |
| ***Analysis and presentation*** |  |
| 14. Show the relation of the marker to standard prognostic variables. | Listed in **Table 1**. |
| 15. Present univariate analyses showing the relation between the marker and outcome, with the estimated effect (e.g. hazard ratio and survival probability). Preferably provide similar analyses for all other variables being analyzed. For the effect of a tumor marker on a time-to-event outcome, a Kaplan-Meier plot is recommended. | Listed in **Table 2**, **Figure 2, Figure 3**, **Figure S1**, and **Figure S2**. |
| 16. For key multivariable analyses, report estimated effects (e.g. hazard ratio) with confidence intervals for the marker and, at least for the final model, all other variables in the model. | Listed in **Table 2.** |
| 17. Among reported results, provide estimated effects with confidence intervals from an analysis in which the marker and standard prognostic variables are included, regardless of their significance. | Listed in **Table 2.** |
| 18. If done, report results of further investigations, such as checking assumptions, sensitivity analyses, and internal validation. | Listed in **Table 3** and **Figure S3**. |
| **Discussion** |  |
| 19. Interpret the results in the context of the pre-specified hypotheses and other relevant studies; include a discussion of limitations of the study. | Details in **Discussion** section. |
| 20. Discuss implications for future research and clinical value. | Details in **Discussion** section. |
